# Supplementary material for: Discovery of an allosteric binding site for anthraquinones at the human P2X4 receptor
Source: Nat Commun. 2025 Dec 4;16:10367. doi: 10.1038/s41467-025-66244-3 (PMC12678798; doi:10.1038/s41467-025-66244-3)
Supplement: Supplementary file 1 — Supplementary Information [file 41467_2025_66244_MOESM1_ESM.pdf]

Supplementary Information

Discovery of an allosteric binding site for anthraquinones at the human P2X4 receptor

Jessica Nagel[1], Vigneshwaran Namasivayam[1], Stephanie Weinhausen[1], Juan Sierra-Marquez[2], Younis Baqi[1,3], Hashem Ali M. Al Musawi[1], Aliaa Abdelrahman[1], Victoria J. Vaaßen[1], Jonathan G. Schlegel[1], Lisa Taplick[1], Jane Torp[4], Jan Kubicek[5], Barbara Maertens[5], Matthias Geyer[4], Tobias Claff[1], Annette Nicke[2], Gregor Hagelueken[4*], and Christa E. Müller[1*]

[1] University of Bonn, PharmaCenter Bonn, Pharmaceutical Institute, Pharmaceutical & Medicinal Chemistry, An der Immenburg 4, 53121 Bonn, Germany

[2] Walther Straub Institute of Pharmacology and Toxicology, Faculty of Medicine, Ludwig-Maximilians-Universität München, Munich, Germany

[3] Department of Chemistry, Faculty of Science, Sultan Qaboos University, PO Box 36, Postal Code 123, Muscat, Oman

[4] University of Bonn, Institute of Structural Biology, Venusberg-Campus 1, 53127 Bonn, Germany

[5] Cube Biotech, Creative Campus Monheim, Creative-Campus-Allee 12, 40789 Monheim, Germany

*Author to whom correspondence should be addressed; Email: christa.mueller@uni-bonn.de, Phone: +49-228-73-2301, ORCID: orcid.org/0000-0002-0013-6624; Email: hagelueken@uni-bonn.de, Phone: +49-228-287-51200, ORCID: orcid.org/0000-0001-8781-5664

Table of Content

Content	Page
**Supplementary Fig. 1** | Structures of selected P2X4 receptor antagonists.	**S3**
**Supplementary Table 1** | Published high-resolution structures of P2X receptors, determined by cryo-EM or X-ray crystallography.	**S4-S8**
**Supplementary Fig. 2** | Protein sequence alignment of the human wt P2X2 with the human wt P2X4 receptor.	**S9**
**Supplementary Fig. 3 |** Concentration-dependent activation of wildtype human P2X4 and P2X2 receptor, and the chimeric P2X4(P2X2) receptors c1, c2-c5, c6, c7, c8, c9, and c10 by ATP.	**S10**
**Supplementary Fig. 4 |** Concentration-dependent activation of P2X4 receptor mutants (D302I, E307T, and Q308T) by ATP.	**S11**
**Supplementary Table 2 |** Potency of ATP at the human wt P2X4 receptor, the human P2X4 receptor mutants, and the chimeric receptor P2X4(P2X2) $c10^{R301\text{-}Q308}$, stably expressed in 1321N1 astrocytoma cells, determined by measurement of calcium influx.	**S12**
**Supplementary Table 3 |** Potency of Cibacron Blue determined in calcium influx assays at the human wt P2X4 receptor, the human wt P2X2 receptor, P2X4 receptor mutants, and the chimeric receptor P2X4(P2X2) $c10^{R301\text{-}Q308}$, stably expressed in 1321N1 astrocytoma cells.	**S13**
**Supplementary Fig. 5 |** Concentration-dependent inhibition (potentiation) of chimeric P2X4(P2X2) receptors $c2\text{-}c5^{C116\text{-}T186}$, $c6^{N208\text{-}S216}$, $c7^{I218\text{-}D224}$, and $c8^{R265\text{-}L269}$ by Cibacron Blue.	**S14**
**Supplementary Table 4** | Cryo-EM data collection, refinement and validation statistics.	**S15**
**Supplementary Fig. 6** | Cryo-EM processing workflow.	**S16**
**Supplementary Fig. 7** | 3D reconstruction of the human P2X4 receptor.	**S17**
**Supplementary Fig. 8** | Comparison of the human P2X4 receptor structure with similar structures.	**S18**
**Supplementary Fig. 9 |** Manual docking of the anthraquinone derivatives PSB-25012 and PSB-0826 into the P2X4-PSB-0704 structure.	**S19**
**Supplementary Fig. 10 |** Dependency of antagonist effects on preincubation time and ATP concentration.	**S20**
**Supplementary Fig. 11 |** Concentration-dependent inhibition of P2X4-E307T receptor mutant by BX430, BAY-1797, and 5-BDBD.	**S20**
**Supplementary Fig. 12 |** AF3 models used in this study with their corresponding statistics.	**S21**
**Supplementary References**	**S21-S22**

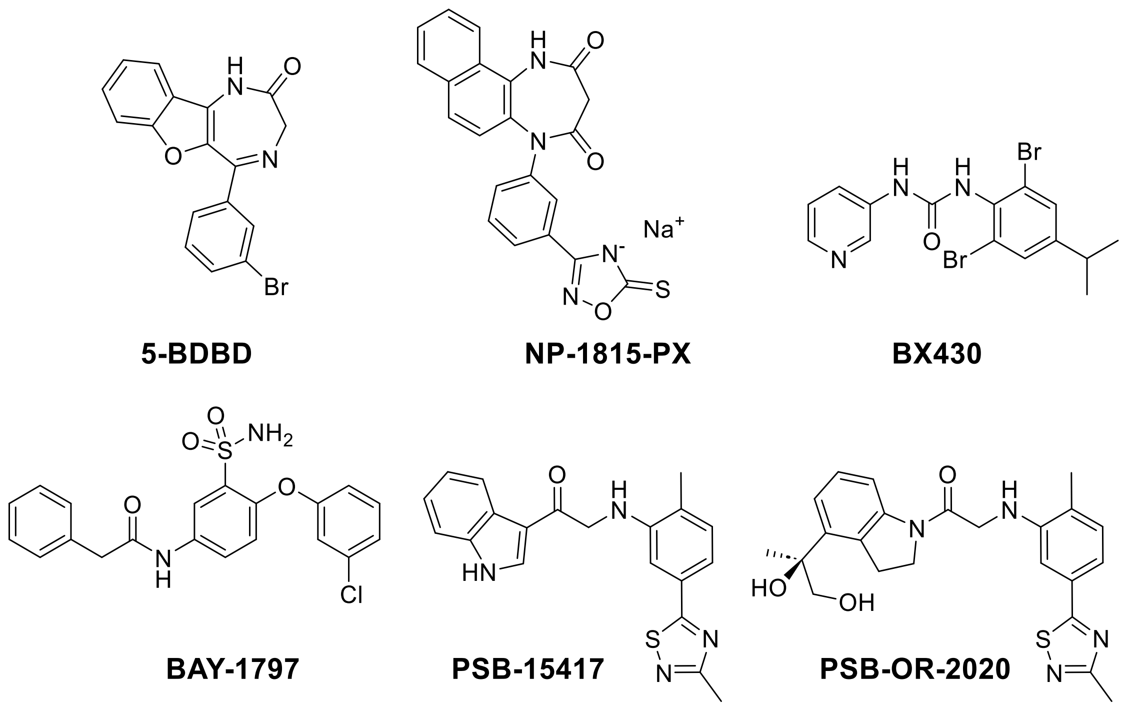

Supplementary Fig. 1 | Structures of selected P2X4 receptor antagonists.

Supplementary Table 1 | Published high-resolution structures of P2X receptors, determined by cryo-EM or X-ray crystallography.

receptor	species	PDB ID	resolution [Å]	state	ligand	truncations	mutations	method	reference
**P2X4**	zebrafish	3i5d<br>3h9v	3.46<br>3.10	closed, apo	/	ΔN27/ΔC8<br>ΔN27/ΔC8	/<br>C51F; N78K; N187R	X-ray	Kawate et al.[1]
	zebrafish	4dw0	2.90	closed, apo	/	ΔN27/ΔC8	C51F; N78K; N187R	X-ray	Hattori et al.[2]
	zebrafish	4dw1	2.80	open	ATP	ΔN27/ΔC24	N78K; N187R	X-ray	Hattori et al.[2]
	zebrafish	8jv5	3.23	closed	BX430	ΔN8/ΔC7	/	cryo-EM	Shen et al.[3]
	zebrafish	8jv6	3.43	closed	BAY-1797	ΔN8/ΔC7	/	cryo-EM	Shen et al.[3]
	zebrafish	5wzy	2.80	open	CTP	ΔN27/ΔC24	N78K; N187R	X-ray	Kasuya et al.[4]
	human	9bqh	2.27	closed, apo	/	full-length	/	cryo-EM	Shi et al.[5]
	human	9c48	2.40	desensitized	ATP	full-length	/	cryo-EM	Shi et al.[5]
	human	9bqi	2.55	closed	BAY-1797	full-length	/	cryo-EM	Shi et al.[5]
**P2X3**	human	5svj<br>5svs<br>5svt	2.98<br>4.03<br>3.79	closed, apo<br>(Mn^{2+} anomalous)<br>(Cs^{+} anomalous)	/	ΔN5/ΔC33	T13P; S15V; V16I	X-ray	Mansoor et al.[6]
	human	5svk	2.77	open	ATP	ΔN5/ΔC33	T13P; S15V; V16I	X-ray	Mansoor et al.[6]
	human	5svl<br>(5svp)	2.90<br>3.30	desensitized<br>(sulfur anomalous)	ATP	ΔN5/ΔC33	/	X-ray	Mansoor et al.[6]
	human	5svm	3.09	desensitized	2-methylthio-ATP	ΔN5/ΔC33	/	X-ray	Mansoor et al.[6]
	human	5svq	3.25	closed	TNP-ATP	ΔN5/ΔC33	T13P; S15V; V16I	X-ray	Mansoor et al.[6]

receptor	species	PDB ID	resolution [Å]	state	ligand	truncations	mutations	method	reference
	human	5svr	3.13	closed	A-317491	ΔN5/ΔC33	T13P; S15V; V16I	X-ray	Mansoor et al.[6]
	human	5yve	3.40	closed	AF-219 (gefapixant)	ΔN5/ΔC33	T13P; S15V; V16I	X-ray	Wang et al.[7]
	human	9ik1	2.61	closed	compound 26a	ΔN5/ΔC33	T13P; S15V; V16I	cryo-EM	Kim et al.[8]
	human	6ah4	3.30	open	ATP, Ca^{2+} ion	ΔN5/ΔC33	T13P; S15V; V16I	X-ray	Li et al.[9]
	human	6ah5	3.82	open	ATP, Mg^{2+} ion	ΔN5/ΔC33	T13P; S15V; V16I	X-ray	Li et al.[9]

**P2X7**	panda	5u1l	3.40	closed, apo	/	Δ1-21/ Δ360-600	N241S; N284S; V35A; R125A; E174K	X-ray	Karasawa et al.[10]
	panda	5u1u	3.60	closed	A740003	Δ1-21/ Δ360-600	N241S; N284S; V35A; R125A; E174K	X-ray	Karasawa et al.[10]
	panda	5u1v	3.40	closed	A804598	Δ1-21/ Δ360-600	N241S; N284S; V35A; R125A; E174K	X-ray	Karasawa et al.[10]
	panda	5u1w	3.50	closed	AZ10606120	Δ1-21/ Δ360-600	N241S; N284S; V35A; R125A; E174K	X-ray	Karasawa et al.[10]
	panda	5u1x	3.20	closed	JNJ47965567	Δ1-21/ Δ360-600	N241S; N284S; V35A; R125A; E174K	X-ray	Karasawa et al.[10]
	panda	5u1y	3.30	closed	GW791343	Δ1-21/ Δ360-600	N241S; N284S; V35A; R125A; E174K	X-ray	Karasawa et al.[10]
	panda	5u2h	3.90	closed	ATP/ A804598	Δ1-21/ Δ360-600	N241S; N284S; V35A; R125A; E174K	X-ray	Karasawa et al.[10]

	panda	8jv8	3.34	closed	PPNDS	Δ1-21/ Δ360-600	N241S; N284S; V35A; R125A; E174K	cryo-EM	Sheng et al.[11]
	panda	8jv7	3.60	closed	PPADS	Δ1-21/ Δ360-600	N241S; N284S; V35A; R125A; E174K	cryo-EM	Sheng et al.[11]
	chicken	5xw6	3.10	closed	TNP-ATP	ΔN27/ΔC214	N190Q	X-ray	Kasuya et al.[12]
	rat	6u9v	2.90	closed, apo	/	full-length	/	cryo-EM	McCarthy et al.[13]
	rat	6u9w	3.30	open	ATP	full-length	/	cryo-EM	McCarthy et al.[13]
	rat	8tr5<br>8v4s	2.53<br>2.49	closed, apo	/	full-length	/	cryo-EM	Oken et al.[14]
**P2X7**	rat	8trj	2.78	open	BzATP	full-length	/	cryo-EM	Oken et al.[14]
	rat	8tr6	2.18	closed	A438079	full-length	/	cryo-EM	Oken et al.[15]
	rat	8tr7	2.53	closed	A839977	full-length	/	cryo-EM	Oken et al.[15]
	rat	8tr8	2.21	closed	AZD9056	full-length	/	cryo-EM	Oken et al.[15]
	rat	8tra	2.41	closed	GSK1482160	full-length	/	cryo-EM	Oken et al.[15]
	rat	8trb	2.36	closed	JNJ47965567	full-length	/	cryo-EM	Oken et al.[15]
	rat	8trk	2.69	closed	methyl blue	full-length	/	cryo-EM	Oken et al.[15]

**P2X1**	human	9b73	1.96	desensitized	ATP	full-length	/	cryo-EM	Bennetts et al.[16]
	human	9b95	2.61	closed	NF449	full-length	/	cryo-EM	Bennetts et al.[16]
	human	9c2a	2.74	closed, apo	/	full-length	/	cryo-EM	Oken et al.[17]
	human	9c2b	2.42	desensitized	ATP	full-length	/	cryo-EM	Oken et al.[17]
	human	9c2c	2.90	closed	NF449	full-length	/	cryo-EM	Oken et al.[17]
**P2X**	gulf coast tick	5f1c	2.90	open	ATP, Zn^{2+} ion	ΔN23/ΔC7	N171Q; C374L	X-ray	Kasuya et al.[18]

```
                                                                         |----TM1---
hP2X2_gi-25092719       MAAAQPKYPAGATARRLARGCW----SALWDYETPKVIVVRNRRLGVLYRAVQLLILLYF  56
hP2X4_gi-116242696      -----------------MAGCCAALAAFLFEYDTPRIVLIRSRKVGLMNRAVQLLILAYV  43
                                           **     : *::*:**::::*.*::*::  ******** *.
                        ------|                     |-----v1----|
hP2X2_gi-25092719       VWYVFIVQKSYQESETGPESSIITKVKGITT-----SEHKVWDVEEYVKPPEGGSVFSII  111
hP2X4_gi-116242696      IGWVFVWEKGYQETDSVV-SSVTTKVKGVAVTNTSKLGFRIWDVADYVIPAQEENSLFVM  102
                        : :**: :*.***:::   **: *****::.       .::*** :** * :  . : ::
                                          |-v2-|       |---v3---|        |--v4-|
hP2X2_gi-25092719       TRVEATHSQTQGTCPESIRVHNATCLSDADCVAGELDMLGNGLRTGRCVPYYQGPSKTCE  171
hP2X4_gi-116242696      TNVILTMNQTQGLCPEIPD-ATTVCKSDASCTAGSAGTHSNGVSTGRCVAF-NGSVKTCE  160
                        *.*  * .**** ***     .:.* ***.*.**. .  .**: ***** : :*  ****
                              |------v5-----|                          |---v6--| |--
hP2X2_gi-25092719       VFGWCPVEDGASVSQF-LGTMAPNFTILIKNSIHYPKFHFSKGNIA-DRTDGYLKRCTFH  229
hP2X4_gi-116242696      VAAWCPVEDDTHVPQPAFLKAAENFTLLVKNNIWYPKFNFSKRNILPNITTTYLKSCIYD  220
                        * .******.: * *  : . * ***:*:**.* ****:*** **  : *  *** * :.
                        -v7|                                        |-v8|
hP2X2_gi-25092719       EASDLYCPIFKLGFIVEKAGESFTELAHKGGVIGVIINWDCDLDLPASECNPKYSFRRLD  289
hP2X4_gi-116242696      AKTDPFCPIFRLGKIVENAGHSFQDMAVEGGIMGIQVNWDCNLDRAASLCLPRYSFRRLD  280
                          :* :****:** ***:**.** ::* :**::*: :****:**  ** * *:*******
                        |---v9---|          |--v10-|                       |--------
hP2X2_gi-25092719       PKHVP--ASSGYNFRFAKYYK-INGTTTRTLIKAYGIRIDVIVHGQAGKFSLIPTIINLA  346
hP2X4_gi-116242696      TRDVEHNVSPGYNFRFAKYYRDLAGNEQRTLIKAYGIRFDIIVFGKAGKFDIIPTMINIG  340
                         :.*   .* **********: : *.  **********:*:**.*:****.:***:**:.
                        ----TM2----|
hP2X2_gi-25092719       TALTSVGVGSFLCDWILLTFMNKNKVYSHKKFDKVCTPSHPSGSWPVTLARVLGQAPPEP  406
hP2X4_gi-116242696      SGLALLGMATVLCDIIVLYCMKKRLYYREKKYKYVEDYEQ-------GLASELDQ-----  388
                        :.*: :*:.:.*** *:*  *:*.  * .**:. *   .:        **  *.*

hP2X2_gi-25092719       GHRSEDQHPSPPSGQEGQQGAECGPAFPPLRPCPISAPSEQMVDTPASEPAQASTPTDPK  466
hP2X4_gi-116242696      ------------------------------------------------------------  388


hP2X2_gi-25092719       GLAQL         471
hP2X4_gi-116242696      -----         388
```

Supplementary Fig. 2 | Protein sequence alignment of the human wt P2X2 with the human wt P2X4 receptor created with Clustal Omega (1.2.4.). Stretches of non-conserved amino acid sequences were identified and labeled as variable region v1 (blue), v2-v5 (pink), v6 (cyan), v7 (green), v8 (orange), v9 (dark green), v10 (red)[19]. The following chimeric (c) receptors were obtained, in which the respective colored sequence of v1-v10 was exchanged for the corresponding P2X2 receptor sequence: c1$^{V71\text{-}I83}$, c2-c5$^{C116\text{-}T186}$, c6$^{N208\text{-}S216}$, c7$^{I218\text{-}D224}$, c8$^{R265\text{-}L269}$, c9$^{T281\text{-}P290}$, and c10$^{R301\text{-}Q308}$, as previously described[19]. Symbols: (*) indicates conserved residues, (:) and (.) indicate conservative and semi-conservative exchanges, and blank spaces point out non-conservative exchanges of residues between aligned protein sequences.

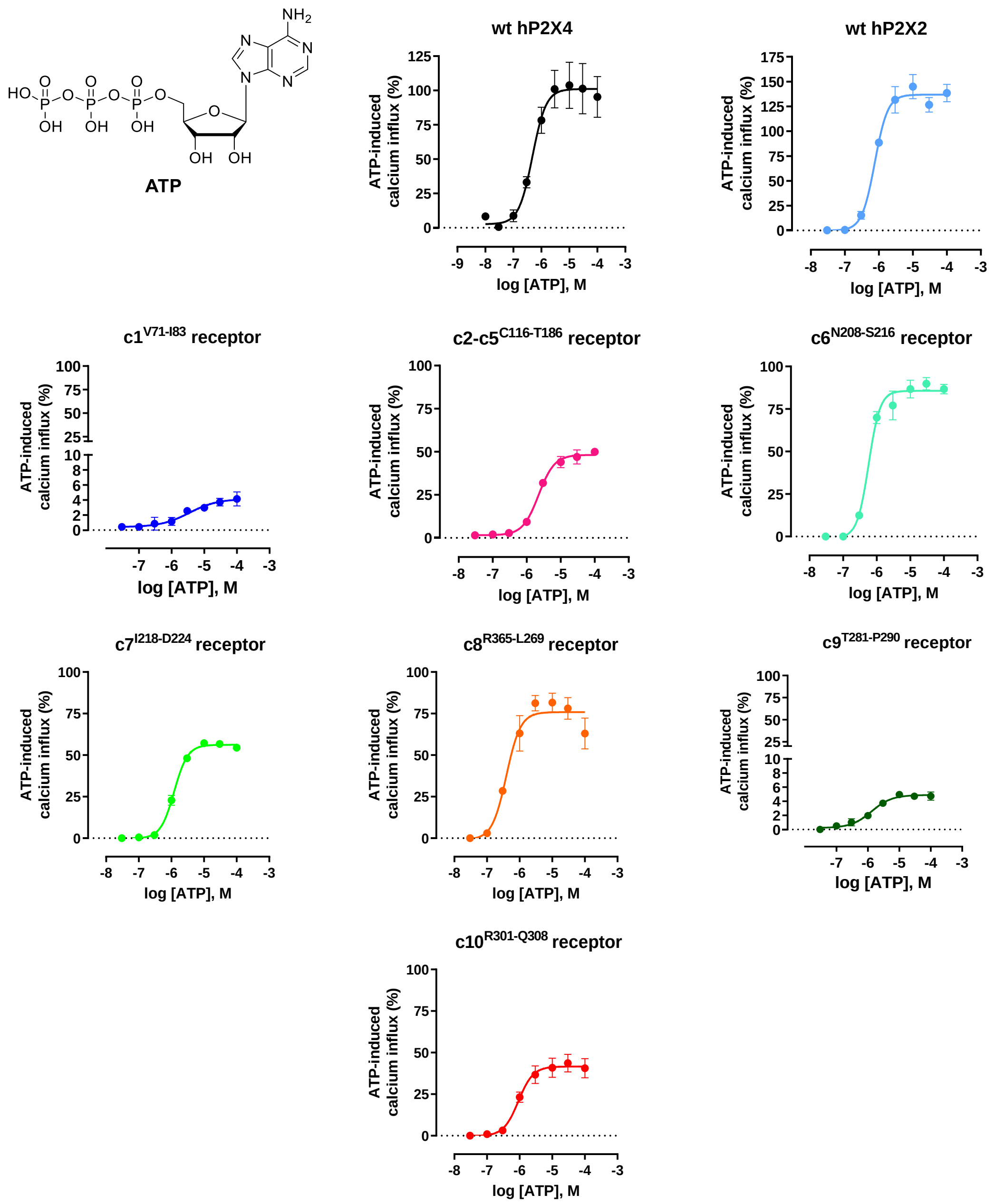

Supplementary Fig. 3 | Concentration-dependent activation of wildtype (wt) human (h) P2X4 and P2X2 receptor, and the chimeric P2X4(P2X2) receptor c1, c2-c5, c6, c7, c8, c9, and c10 by ATP[19]. Receptors were stably expressed in 1321N1 astrocytoma cells. ATP-dependent receptor activation was measured in calcium influx assays. Data was normalized to the maximal ATP-induced effect (100%). Data represent means ± SEM of at least three independent biological replicates performed in technical duplicates. Source data are provided as a Source Data file.

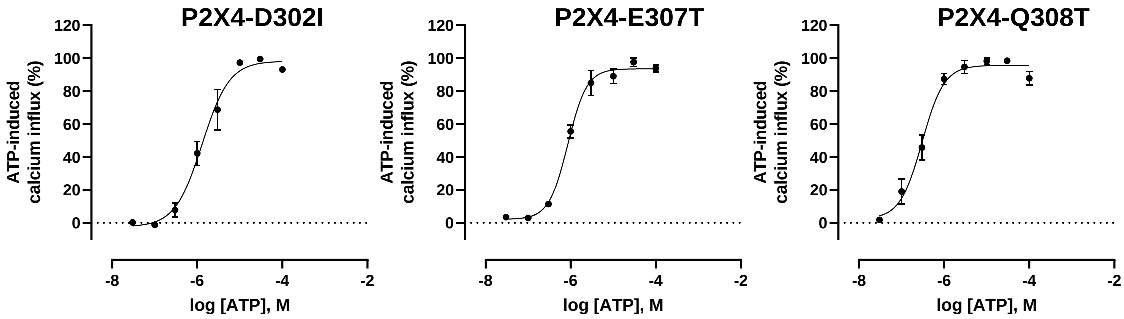

Supplementary Fig. 4 | Concentration-dependent activation of P2X4 receptor mutants (D302I, E307T, and Q308T) by ATP. Receptors were stably expressed in 1321N1 astrocytoma cells. ATP-dependent receptor activation was measured in calcium influx assays. Data was normalized to the maximal ATP-induced effect (100%). Data represent means ± SEM of at least three independent biological replicates performed in technical duplicates. Source data are provided as a Source Data file.

Supplementary Table 2 | Potency of ATP at the wildtype (wt) human (h) P2X4 and P2X2 receptor, and the chimeric P2X4(P2X2) receptors c2-c5$^{C116\text{-}T186}$, c6$^{N208\text{-}S216}$, c7$^{I218\text{-}D224}$, c8$^{R265\text{-}L269}$, c10$^{R301\text{-}Q308}$, and P2X4 receptor mutants (D302I, E307T, Q308T), stably expressed in 1321N1 astrocytoma cells, determined by measurement of calcium influx.

	ATP EC_{50} (µM)[a]	ATP pEC_{50} ± SEM[c]	Adjusted P values[d]
wt hP2X4	0.357	6.45 ± 0.04	/
wt hP2X2	0.637	6.12 ± 0.05	/
P2X4(P2X2) c2-c5$^{C116\text{-}T186}$	2.57[b]	5.64 ± 0.01****	<0.0001
P2X4(P2X2) c6$^{N208\text{-}S216}$	0.572[b]	6.23 ± 0.02**	0.0011
P2X4(P2X2) c7$^{I218\text{-}D224}$	1.14[b]	5.92 ± 0.04****	<0.0001
P2X4(P2X2) c8$^{R265\text{-}L269}$	0.298[b]	6.41 ± 0.04^{ns}	0.8107
P2X4(P2X2) c10$^{R301\text{-}Q308}$	0.862[b]	6.04 ± 0.03****	<0.0001
P2X4-D302I	1.60	5.85 ± 0.15**	0.0046
P2X4-E307T	0.921	6.04 ± 0.03*	0.0334
P2X4-Q308T	0.295	6.56 ± 0.10^{ns}	0.7694

[a]Data represent mean EC_{50} values of ATP obtained in three biological replicates performed in technical duplicates. [b]Published values[19]. [c]Data represent pEC_{50} values ± SEM. The level of significance was calculated by a one-way ANOVA with Dunnett's multiple comparisons test. Significance was expected if P value was ≤ 0.05. The significance levels were defined as follows: * $P < 0.05$, ** $P < 0.01$, *** $P < 0.001$, **** $P < 0.0001$; ns, not significant. [d]Adjusted P values are presented. The pEC_{50} values of ATP at the chimeric receptors and at the P2X4 receptor mutants were compared to the pEC_{50} value at the wt hP2X4 receptor, respectively.

Supplementary Table 3 | Potency of Cibacron Blue at the wildtype (wt) human (h) P2X4 and P2X2 receptor, and the chimeric P2X4(P2X2) receptors c2-c5$^{C116\text{-}T186}$, c6$^{N208\text{-}S216}$, c7$^{I218\text{-}D224}$, c8$^{R265\text{-}L269}$, c10$^{R301\text{-}Q308}$, and P2X4 receptor mutants (D302I, E307T, Q308T), stably expressed in 1321N1 astrocytoma cells, determined by measurement of calcium influx.

	Cibacron Blue IC$_{50}$ (µM)[a]	Cibacron Blue pIC$_{50}$ ± SEM (n value)[b]	Adjusted P values[c]
wt hP2X4	12.1 (EC$_{50}$: 0.243)[d]	4.96 ± 0.14 (3)	
wt hP2X2	13.1	4.90 ± 0.07^{ns} (3)	0.9971
P2X4(P2X2) c2-c5$^{C116\text{-}T186}$	8.99	5.05 ± 0.06^{ns} (2)	0.9933
P2X4(P2X2) c6$^{N208\text{-}S216}$	8.60 (EC$_{50}$: 0.530)[d]	5.07 ± 0.03^{ns} (3)	0.9657
P2X4(P2X2) c7$^{I218\text{-}D224}$	23.6	4.63 ± 0.03^{ns} (3)	0.0835
P2X4(P2X2) c8$^{R265\text{-}L269}$	20.3	4.75 ± 0.16^{ns} (3)	0.4827
P2X4(P2X2) c10$^{R301\text{-}Q308}$	0.00476	8.29 ± 0.07**** (5)	<0.0001
P2X4-D302I	1.42	5.85 ± 0.05**** (3)	<0.0001
P2X4-E307T	0.0178	7.78 ± 0.12**** (3)	<0.0001
P2X4-Q308T	10.4	5.00 ± 0.06^{ns} (3)	0.9996

[a]Data represent mean IC$_{50}$ values in µM. The inhibition of Cibacron Blue was determined in the presence of ATP at its respective EC$_{80}$. [b]Data represent pIC$_{50}$ values ± SEM with the number of experiments indicated in parentheses. The level of significance was calculated by a one-way ANOVA with Dunnett's multiple comparisons test. Significance was expected if P value was ≤ 0.05. The significance levels were defined as follows: * $P < 0.05$, ** $P < 0.01$, *** $P < 0.001$, **** $P < 0.0001$; ns, not significant. [c]Adjusted P values are presented. The pIC$_{50}$ values of Cibacron Blue at the receptor mutants were compared to those at the wt P2X4 receptor. [d]Biphasic modulation by Cibacron Blue.

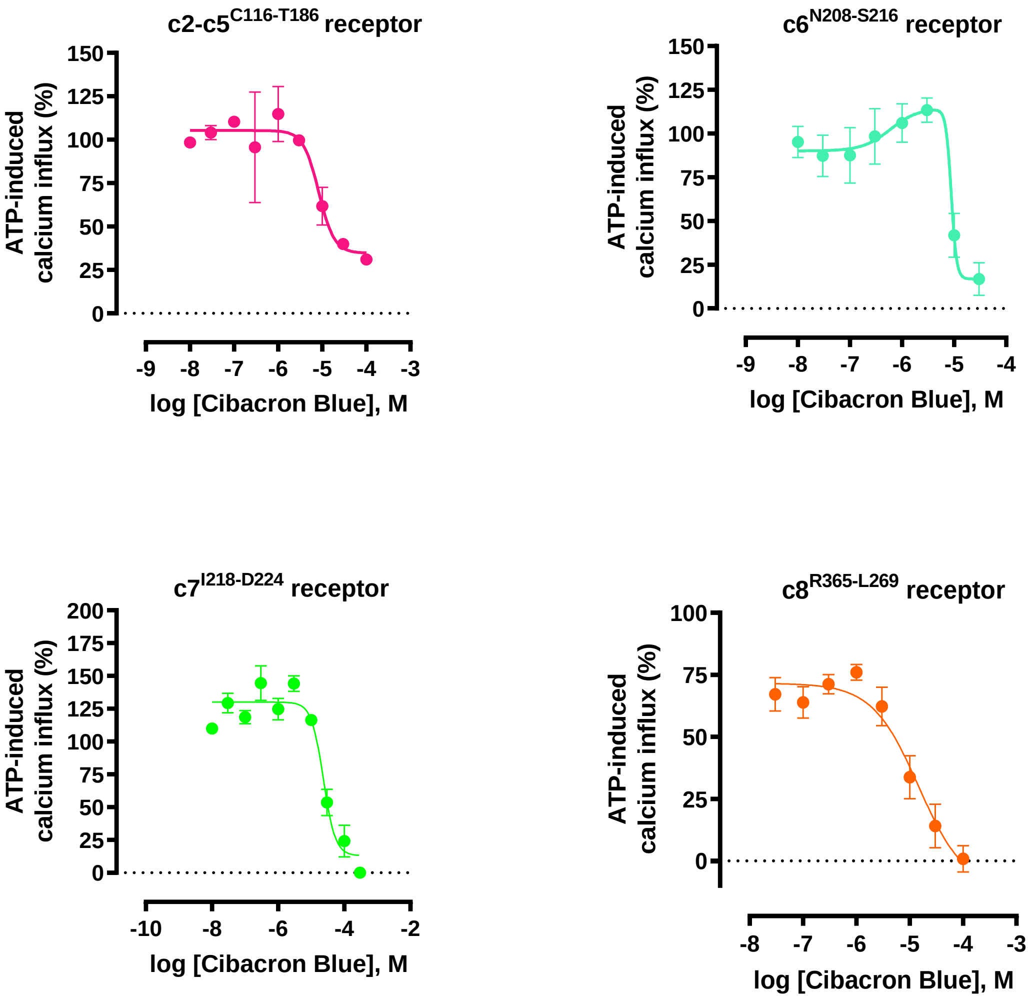

Supplementary Fig. 5 | Concentration-dependent inhibition (potentiation) of chimeric P2X4(P2X2) receptors c2-c5$^{C116\text{-}T186}$, c6$^{N208\text{-}S216}$, c7$^{I218\text{-}D224}$, and c8$^{R265\text{-}L269}$ by Cibacron Blue. Receptors were stably expressed in 1321N1 astrocytoma cells. The effect of Cibacron Blue was determined in the presence of ATP at its respective EC_{80}. Data represent means ± SEM of 2-3 independent biological replicates performed in technical duplicates. Source data are provided as a Source Data file.

Supplementary Table 4 | Cryo-EM data collection, refinement and validation statistics.

	EMDB-51502 (PDB 9GP7)	
**Data collection and processing**		
	focused map	overall map
Magnification	45,000x	
Voltage (kV)	200	
Electron exposure (e–/Å^2)	46.7	
Defocus range (μm)	1.0-2.5	
Pixel size (Å)	0.901	
Symmetry imposed	C3	
Initial particle images (no.)	3,644,764	
Final particle images (no.)	79,284	38,591
Map resolution (Å)	3.35	3.67
FSC threshold	0.143	0.143
Map sharpening B factor	151	167
Map resolution range (Å)	12.2-2.5	13.9-2.5
**Refinement**		
Initial model used (PDB code)	AF-Q99571-F1	
Model composition		
Non-hydrogen atoms	7341 (3x2447)	
Protein residues	909 (3x303)	
Ligands	3 (3x1)	
B factors (Å^2)		
Protein (min/max/avg)	128/226/157	
Ligand (min/max/avg)	137/183/160	
R.m.s. deviations		
Bond lengths (Å)	0.014	
Bond angles (°)	1.863	
Validation		
MolProbity score	1.71	
Clashscore	4.02	
Poor rotamers (%)	0.4	
Ramachandran plot		
Favored (%)	91.0	
Allowed (%)	7.8	
Disallowed (%)	1.3	

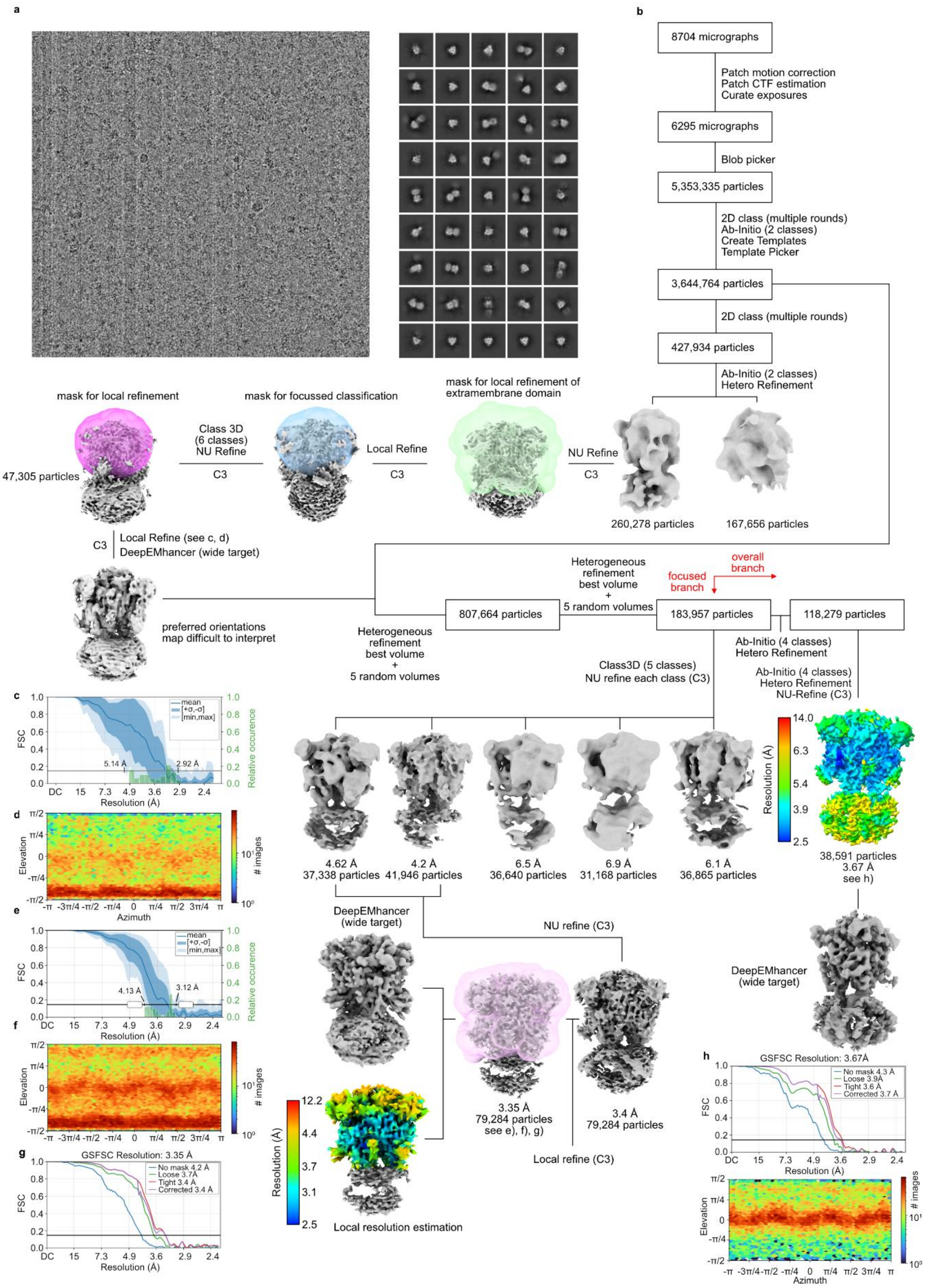

Supplementary Fig. 6 | Cryo-EM processing workflow. a Representative motion corrected micrograph and 2D classes. **b** Schematic of 3D reconstruction workflow. **c** Conical FSC plot revealing severe resolution anisotropy. **d** Viewing angle distribution showing an overrepresentation of "bottom-side" views. **e** Conical FSC plot after skipping 2D classifications as indicated in the processing tree. **f** Viewing angle distribution after skipping 2D classifications. **g** FSC plot of final focused reconstruction. **h** FSC plot of final overall reconstruction and its viewing angle distribution.

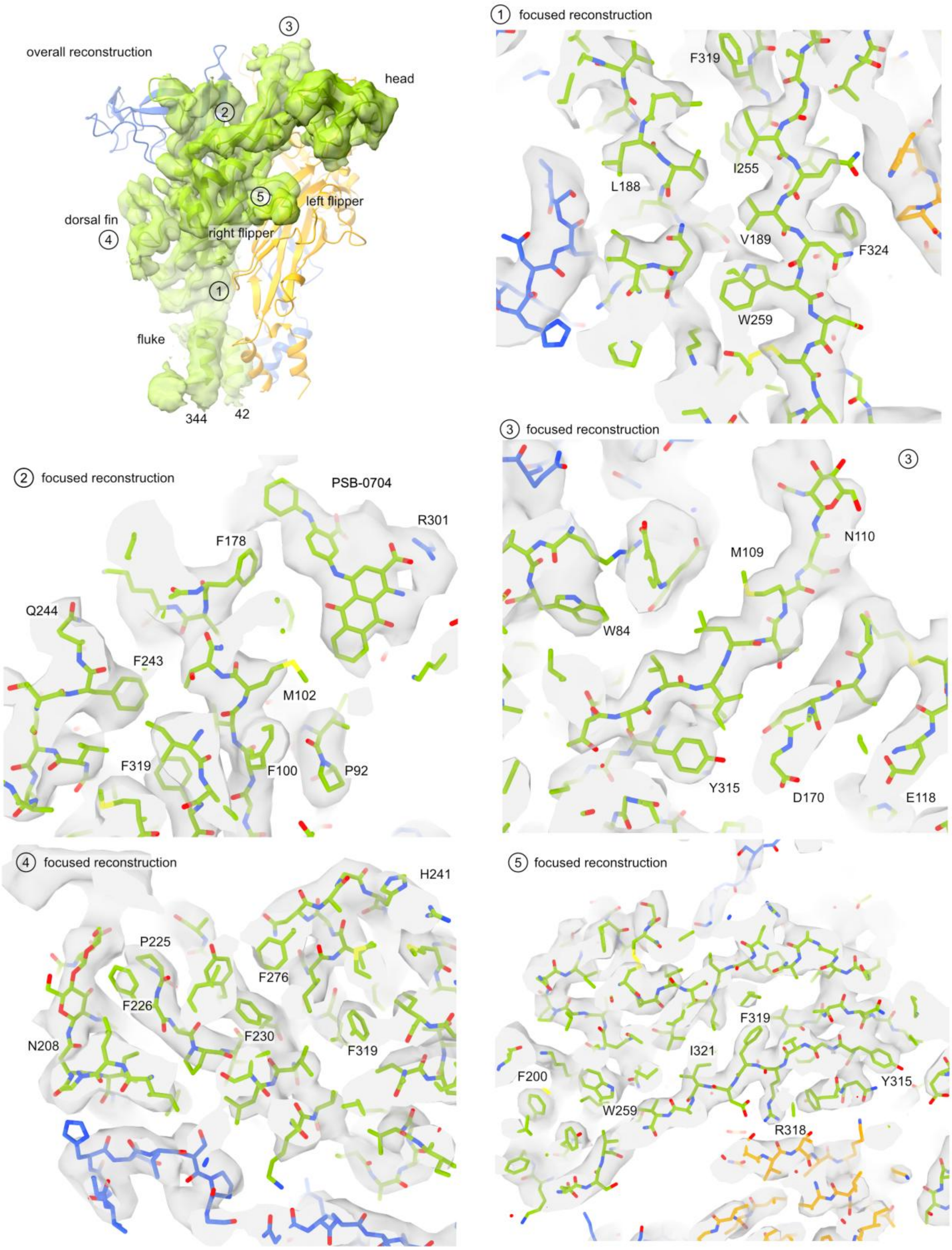

Supplementary Fig. 7 | 3D reconstruction of the human P2X4 receptor. The top left panel shows one chain of the receptor, overlaid on the overall reconstruction and selected regions are indicated by numbers in circles. The remaining panels show snapshots of the focused 3D reconstruction of these regions (gray meshes). Selected residue numbers are indicated.

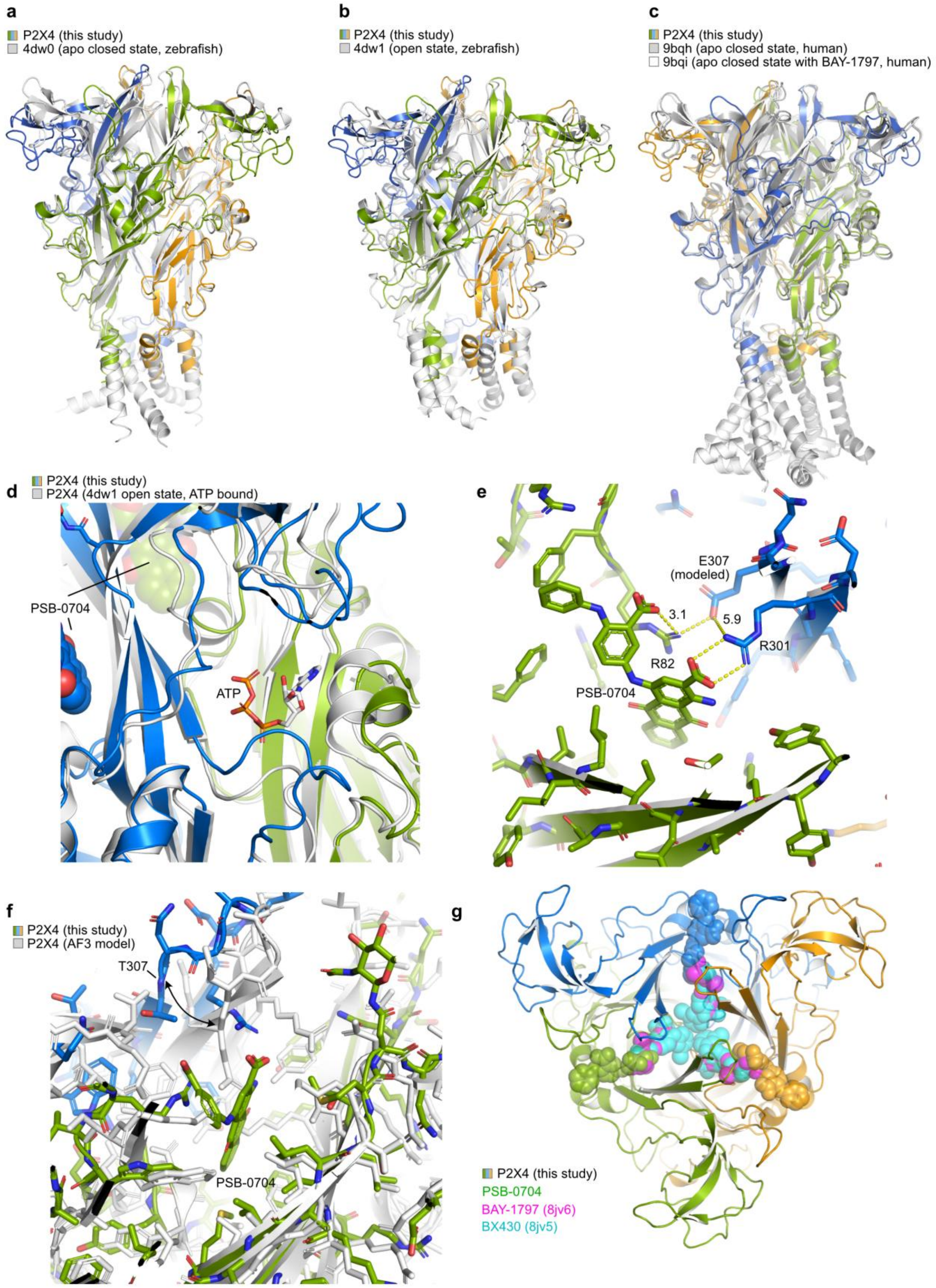

Supplementary Fig. 8 | Comparison of the human P2X4 receptor structure with similar structures. a Superposition of the human P2X4 receptor structure (cartoon, color-coded as in the main text) with the apo closed-state structure (white cartoon) of the zebrafish ortholog; **b** as in (**a**) but with the open-state structure of the zebrafish ortholog; **c** as in (a) but with the desensitized state structure of the human P2X4 receptor. **d** Detail of (b) focused on the ATP-

binding site. The ATP in 4dw1 (white) is shown as sticks, and the ligand PSB-0704 is shown as spheres. **e** Model of PSB-0704 binding to the wt human P2X4 receptor, based on the human P2X4-E307T receptor structure from this work. The threonine at position 307 was replaced by a glutamate residue in PyMOL. The rotamer of the side chain was chosen in such a way that no severe clashes occurred. **f** Cryo-EM structure of the human P2X4-E307T receptor (this work) superimposed with the AF3 model shown in Fig. 2 (white). **g** Cartoon model of the human P2X4 receptor. The antagonist PSB-0704 is shown as green spheres model. The relative positions of the antagonists BAY-1797 and BX430 are indicated. Note that alignments from the foldseek[20] server were used to create the superposition.

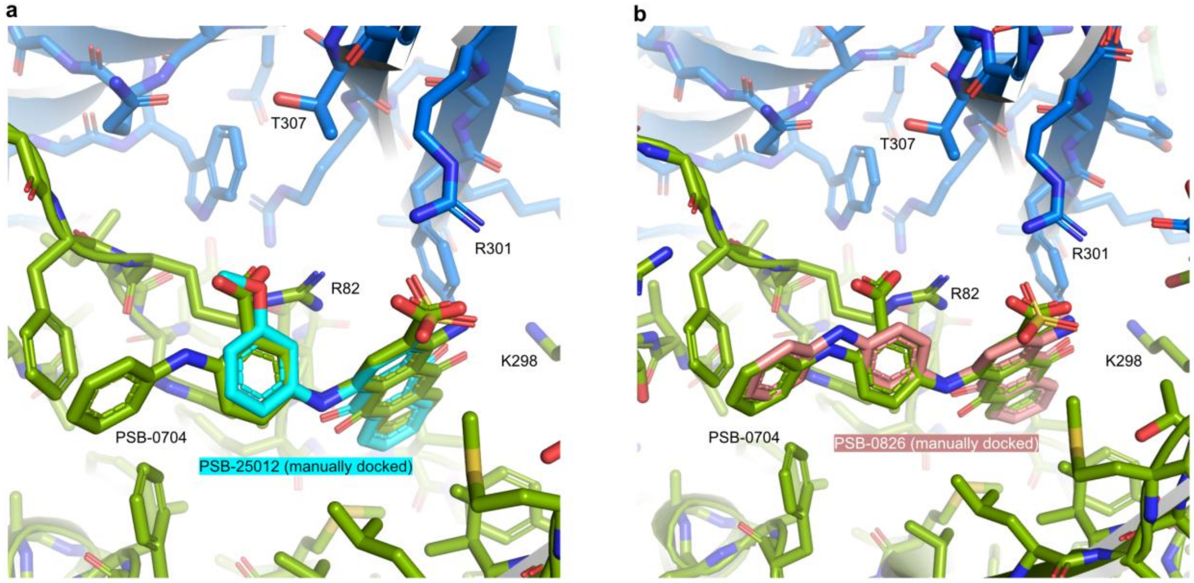

Supplementary Fig. 9 | Manual docking of the anthraquinone derivatives (**a**) PSB-25012 and (**b**) PSB-0826 into the P2X4-PSB-0704 structure. Both compounds showed high inhibitory potency at the human wt P2X4 receptor. Note that these compounds do not have a negatively charged substituent on ring D.

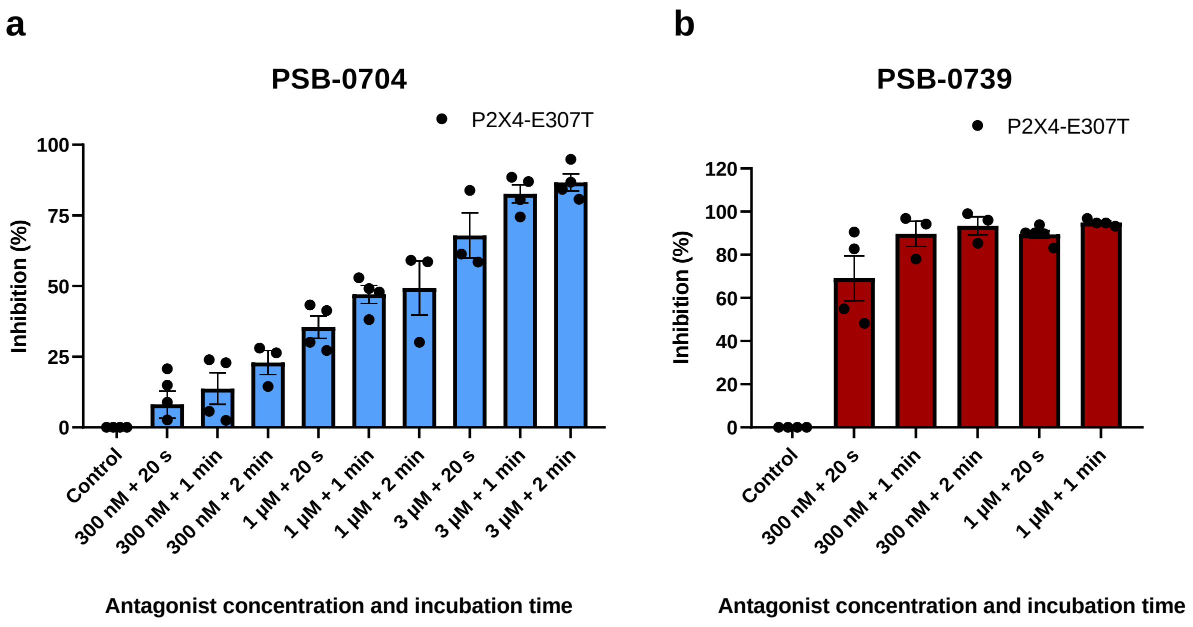

Supplementary Fig. 10 | Dependency of antagonist effects on preincubation time and ATP concentration. The human wt P2X4 and the P2X4-E307T mutant were expressed in *Xenopus laevis* oocytes and analyzed by TEVC at – 60 mV. Inhibitory effects of PSB-0704 (**a**) and PSB-0739 (**b**) after superfusion with the antagonists at the indicated concentrations and incubation times. Current responses were evoked by 3 s-pulses of 10 mM ATP in the absence and presence of antagonist. Data are presented as mean ± SEM from at least 3 oocytes and represented as percentage of inhibition relative to the preceeding ATP response in the absence of antagonist. Source data are provided as a Source Data file.

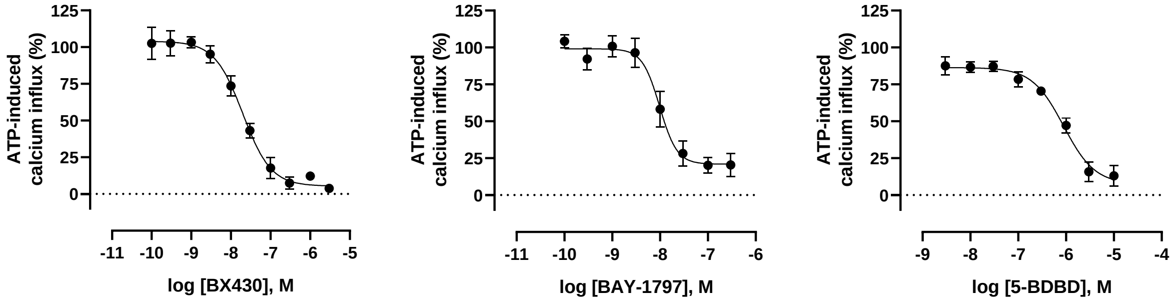

Supplementary Fig. 11 | Concentration-dependent inhibition of P2X4-E307T receptor mutant by BX430, BAY-1797, and 5-BDBD in the presence of ATP at its respective EC_{80}. Data represent means ± SEM of at least three biological replicates performed in technical duplicates. Source data are provided as a Source Data file.

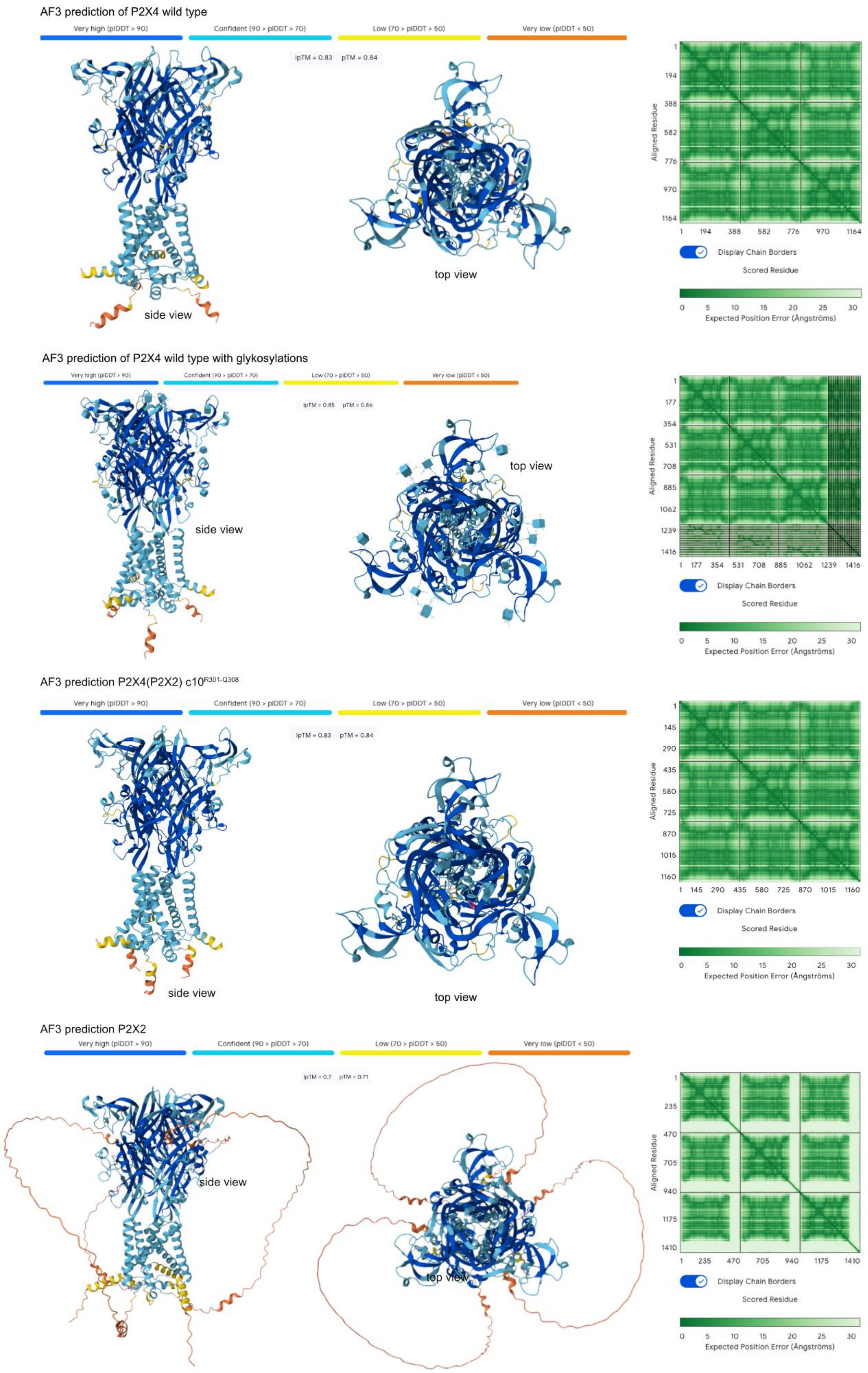

Supplementary Fig. 12 | AF3 (https://alphafoldserver.com/) models used in this study with their corresponding statistics. For each model, two views are shown and the structures are colored according to their pLDDT (predicted local distance difference test). The models, including their sequences and all output of the AF3 server are provided as source data.

Supplementary References

1. Kawate, T., Michel, J. C., Birdsong, W. T. & Gouaux, E. Crystal structure of the ATP-gated P2X4 ion channel in the closed state. *Nature* **460**, 592–598 (2009).
2. Hattori, M. & Gouaux, E. Molecular mechanism of ATP binding and ion channel activation in P2X receptors. *Nature* **485**, 207–212 (2012).
3. Shen, C., Zhang, Y., Cui, W., Zhao, Y., Sheng, D., Teng, X., Shao, M., Ichikawa, M., Wang, J. & Hattori, M. Structural insights into the allosteric inhibition of P2X4 receptors. *Nat. Commun.* **14**, 6437 (2023).
4. Kasuya, G., Fujiwara, Y., Tsukamoto, H., Morinaga, S., Ryu, S., Touhara, K., Ishitani, R., Furutani, Y., Hattori, M. & Nureki, O. Structural insights into the nucleotide base specificity of P2X receptors. *Sci. Rep.* **7**, 45208 (2017).
5. Shi, H., Ditter, I. A., Oken, A. C. & Mansoor, S. E. Human P2X4 receptor gating is modulated by a stable cytoplasmic cap and a unique allosteric pocket. *Sci. Adv.* **11**, eadr3315 (2025).
6. Mansoor, S. E., Lü, W., Oosterheert, W., Shekhar, M., Tajkhorshid, E. & Gouaux, E. X-ray structures define human P2X3 receptor gating cycle and antagonist action. *Nature* **538**, 66–71 (2016).
7. Wang, J., Wang, Y., Cui, W.-W., Huang, Y., Yang, Y., Liu, Y., Zhao, W.-S., Cheng, X.-Y., Sun, W.-S., Cao, P., Zhu, M. X., Wang, R., Hattori, M. & Yu, Y. Druggable negative allosteric site of P2X3 receptors. *Proc. Natl. Acad. Sci. USA* **115**, 4939–4944 (2018).
8. Kim, G.-R., Kim, S., Kim, Y.-O., Han, X., Nagel, J., Kim, J., Song, D. I., Müller, C. E., Yoon, M.-H., Jin, M. S. & Kim, Y.-C. Discovery of triazolopyrimidine derivatives as selective P2X3 receptor antagonists binding to an unprecedented allosteric site as evidenced by cryo-electron microscopy. *J. Med. Chem.* **67**, 14443–14465 (2024).
9. Li, M., Wang, Y., Banerjee, R., Marinelli, F., Silberberg, S., Faraldo-Gómez, J. D., Hattori, M. & Swartz, K. J. Molecular mechanisms of human P2X3 receptor channel activation and modulation by divalent cation bound ATP. *Elife* **8**, e47060 (2019).
10. Karasawa, A. & Kawate, T. Structural basis for subtype-specific inhibition of the P2X7 receptor. *Elife* **5**, e22153 (2016).
11. Sheng, D., Yue, C., Jin, F., Wang, Y., Ichikawa, M., Yu, Y., Guo, C.-R. & Hattori, M. Structural insights into the orthosteric inhibition of P2X receptors by non-ATP-analog antagonists. *Elife* **12**, RP92829 (2024).

12. Kasuya, G., Yamaura, T., Ma, X.-B., Nakamura, R., Takemoto, M., Nagumo, H., Tanaka, E., Dohmae, N., Nakane, T., Yu, Y., Ishitani, R., Matsuzaki, O., Hattori, M. & Nureki, O. Structural insights into the competitive inhibition of the ATP-gated P2X receptor channel. *Nat. Commun.* **8**, 876 (2017).
13. McCarthy, A. E., Yoshioka, C. & Mansoor, S. E. Full-length P2X7 structures reveal how palmitoylation prevents channel desensitization. *Cell* **179**, 659-670.e13 (2019).
14. Oken, A. C., Lisi, N. E., Krishnamurthy, I., McCarthy, A. E., Godsey, M. H., Glasfeld, A. & Mansoor, S. E. High-affinity agonism at the P2X7 receptor is mediated by three residues outside the orthosteric pocket. *Nat. Commun.* **15**, 6662 (2024).
15. Oken, A. C., Ditter, I. A., Lisi, N. E., Krishnamurthy, I., Godsey, M. H. & Mansoor, S. E. P2X7 receptors exhibit at least three modes of allosteric antagonism. *Sci. Adv.* **10** (2024).
16. Bennetts, F. M., Venugopal, H., Glukhova, A., Mobbs, J. I., Ventura, S. & Thal, D. M. Structural insights into the human P2X1 receptor and ligand interactions. *Nat. Commun.* **15** (2024).
17. Oken, A. C., Lisi, N. E., Ditter, I. A., Shi, H., Nechiporuk, N. A. & Mansoor, S. E. Cryo-EM structures of the human P2X1 receptor reveal subtype-specific architecture and antagonism by supramolecular ligand-binding. *Nat. Commun.* **15** (2024).
18. Kasuya, G., Fujiwara, Y., Takemoto, M., Dohmae, N., Nakada-Nakura, Y., Ishitani, R., Hattori, M. & Nureki, O. Structural insights into divalent cation modulations of ATP-gated P2X receptor channels. *Cell Rep.* **14**, 932–944 (2016).
19. Weinhausen, S., Nagel, J., Namasivayam, V., Spanier, C., Abdelrahman, A., Hanck, T., Hausmann, R. & Müller, C. E. Extracellular binding sites of positive and negative allosteric P2X4 receptor modulators. *Life Sci.* **311**, 121143 (2022).
20. Van Kempen, M., Kim, S. S., Tumescheit, C., Mirdita, M., Lee, J., Gilchrist, C.L.M., Söding, J., Steinegger, M. Fast and accurate protein structure search with Foldseek. *Nat. Biotechnol.* **42**, 243–246 (2024).
